# Integrating genomic predictions into an applied Central European wheat breeding program

**DOI:** 10.1007/s00122-026-05175-z

**Published:** 2026-02-13

**Authors:** Lars Erik Thomsen, Yusheng Zhao, Ulrike Avenhaus, Jochen Christoph Reif, Ravindra Reddy Gundala

**Affiliations:** 1 Department of Breeding Research, Leibniz Institute of Plant Genetics and Crop Plant Research (IPK), Corrensstraße 3 OT Gatersleben, 06466 Seeland, Germany; 2 W. von Borries-Eckendorf GmbH & Co. KG, Hovedisser Straße 94, 33818 Leopoldshöhe, Germany; 3 Present Address: Department of Biometry and Population Genetics, Justus Liebig University Gießen, Heinrich-Buff-Ring 26, 35392 Gießen, Germany

## Abstract

**Key message:**

Genomic prediction is most effective when balancing heritability and population size; advanced breeding stages suit complex traits, broader training sets improve predictions in simple traits—enabling efficient, earlier selection.

**Abstract:**

Genomic selection can reduce generation intervals and costs, thereby increasing genetic gain in plant breeding. The study aimed to assess the impact of phenotypic data quality, sample size, and diversity across different breeding stages on the accuracy of genomic predictions. We used phenotypic data of the Central European winter bread wheat breeding company W. von Borries-Eckendorf GmbH & Co. KG, comprising 13,773 genotypes evaluated in up to 95 environments in nearly 57,000 plots for grain yield, plant height, protein content and yellow rust resistance. Genotypic data for 6,228 genotypes were generated using a 7,000 SNP Illumina Infinium assay. We implemented genomic best linear unbiased prediction and tested prediction ability within and across breeding stages. Three prediction scenarios were evaluated to examine the prediction ability: (1) within individual breeding stages, (2) across breeding stages, and (3) for most advanced genotypes. Results indicate more accurate models trained on mid- or late-stage phenotypic data for most traits compared to early-stage data. Combining mid- and late-stage data further improved predictions for complex traits like grain yield and yellow rust resistance. These findings highlight the importance of balancing high heritability and appropriate training sets for optimizing genomic predictions, demonstrating the potential of genomic selection as a cornerstone for wheat breeding programs.

**Supplementary Information:**

The online version contains supplementary material available at 10.1007/s00122-026-05175-z.

## Introduction

The trajectory of global population growth is anticipated to continue at a similar or even faster pace in the coming decades (FAOSTAT 2024). A systematic literature review by Van Dijk et al. (2021) on future food security predicts a 35–56% increase in global food demand compared to 2010, highlighting the ongoing socioeconomic risk of hunger for millions of people. Among all cultivated crops, wheat (*Triticum aestivum* L.) is crucial in enhancing global food security, contributing around 19% of caloric and 21% of protein intake (Shiferaw et al. 2013). As many of the improvements in wheat productivity are results of breeding advances (Laidig et al. 2014; Laidig et al. 2017; Voss-Fels et al. 2019), accelerating selection gain in wheat breeding will be crucial to meet food security in the future.

Further, genomic selection is proposed to be an efficient strategy to multiply the genetic gain per unit time in breeding programs (Tessema et al. 2020). Multiple experimental studies support its use in plant breeding (e.g., Rutkoski et al. 2015; Battenfield et al. 2016; He et al. 2016; Michael et al. 2018; Dreisigacker et al. 2023; El Hanafi et al. 2023; Ficht et al. 2023). In genomic prediction, an individual’s genetic value is predicted based on genetic markers. The effects of these markers must be estimated in advance using a representative and comprehensive training population. The successful implementation of genomic selection relies heavily on the accuracy of the genomic prediction, measured as the correlation between predicted and observed values. Because this so-called prediction ability is directly linked to the genetic gain in the breeder’s equation, increasing the prediction ability is crucial for accelerating the genetic gain (Falconer and Mackay 1996).

However, numerous interrelated factors influence the prediction ability in genomic predictions, including the training set size (VanRaden et al. 2009; Akdemir et al. 2015; Edwards et al. 2019), population structure (Windhausen et al. 2012; Rincent et al. 2017), relatedness between the training set and the test set (Isidro et al. 2015; Neyhart et al. 2017; Lozada et al. 2019), heritability of the phenotypic data (Hayes et al. 2009; Liu et al. 2018), and marker density (Daetwyler et al. 2010). While these factors have been explored in theoretical and experimental settings, there is limited information on how to apply routine breeding data for genomic predictions in commercial wheat breeding programs, where resource allocation, data quality, and stage-specific selection differ markedly.

As a standard practice in breeding programs, the allocation of resources is adjusted from testing a large number of entries with no replications in fewer environments during early breeding stages to testing a reduced number of entries with replications in more environments during later and more advanced breeding stages, where cultivar release decisions become more critical. This intensification of field tests influences phenotypic data quality and precision of genotypic value estimation, which further influences the prediction ability if training sets are not carefully composed (Bernardo 2014). For example, He et al. (2016) studied the composition of a training set for a commercial winter wheat breeding program and suggested the benefits of excluding data from early breeding stages. However, their study used limited phenotypic data from two years and focused exclusively on grain yield. Therefore, it is essential to establish clear guidelines for constructing training sets that effectively integrate data from different breeding stages, with the aim of enhancing prediction ability across traits of varying genetic complexity.

In this study, therefore, we explored the opportunities to incorporate genomic predictions and translate genomic predictions into routine decision-making in large-scale breeding programs. This study explores prediction scenarios within an applied winter wheat breeding program, focusing on data for grain yield, plant height, protein content, and yellow rust resistance. We analyzed data from three breeding stages, each representing different data quality and quantity, to evaluate their suitability for genomic predictions. This study’s objectives were to (i) curate a comprehensive set of phenotypic and genotypic data generated in the course of a commercial winter wheat breeding program and evaluate the resulting data quality, (ii) examine prediction abilities using different combinations of early-, mid-, and late-stage training and test populations, and (iii) provide recommendations for the breeder on compilation of optimum training populations for genomic predictions.

## Material and methods

### Plant material and field trials

The germplasm analyzed in this study was developed through the winter wheat breeding program of W. von Borries-Eckendorf GmbH & Co. KG (WvB). The genotypes are elite breeding lines selected for their suitability for wheat production in Central Europe. The study includes genotypes from the final three breeding stages before candidates enter official variety testing, designated as WP-3, WP-2, and WP-1. The number in the suffix corresponds to the number of years remaining in the breeding pipeline before the genotypes are submitted for registration at the Federal Plant Variety Office.

Phenotypic data for around 2,500 genotypes per year from the WP-3 stage were available and tested in field trials at a single treated location. These treated trials involved the application of fertilizers, fungicides, and growth regulators but were not replicated, except for the inclusion of intensively replicated check varieties in every tenth plot. For the WP-2 stage, phenotypic data were available for approximately 500 genotypes annually, evaluated across up to four treated locations (replicated) and seven untreated locations (unreplicated and without the application of fungicides and growth regulators). In the most advanced stage, WP-1, evaluations for about 75 genotypes per year were possible based on field trials across twelve treated and seven untreated locations. The field evaluations for WP-1 and WP-2 in treated trials were based on partial replicated designs. The untreated trials were conducted without replication at each location. Overall, phenotypic data were available for 16,300 plots in WP-1, 29,400 plots in WP-2, and 11,100 plots in WP-3. In total, the study incorporates phenotypic data from 95 distinct environments, defined not only as combinations of locations (up to 17) and years (from 2019 to 2023) but also distinguished between treated and untreated trials. Plot sizes varied between 6.3 m^2^ and 12 m^2^. A schematic overview of the breeding pipeline, showing the flow of genotypes from WP-3 to WP-1 and the associated changes in testing intensity and population size, is provided in Supplementary Fig. 1. An overview of the number of environments, experimental designs, and traits assessed across breeding stages is provided in Supplementary Table 1.

Phenotypic data for grain yield, plant height, and protein content were recorded in treated trials, while resistance to yellow rust caused by *Puccinia striiformis* f.sp. *tritici* was assessed in untreated trials. Grain yield was determined by weighing the harvested grain using a combine harvester. A 100 g seed sample was collected from each plot during harvest and analyzed for seed moisture content and protein content with near-infrared reflection spectroscopy (Perten Instruments, Model DA 7200, Springfield; IL, USA). Seed moisture content was used to adjust the weight of the harvested grain to a relative moisture content of 14.0%. These corrected grain yield values were then converted to a yield in quintals per hectare (q.ha^−1^). For plant height, the average length of the main wheat shoots was measured from representative plants located in the center of each plot. Following the official protocols of the German Federal Plant Variety Office, a scoring scale from 1 (fully resistant) to 9 (extremely susceptible) was used to assess yellow rust resistance (BSA 2000).

### Phenotypic data analysis

Grain yield, plant height, and protein content from untreated trials were excluded from further calculations, as they do not correspond to the same trait in treated trials, as per the definition of the official variety testing in Germany. Yellow rust resistance notations from the WP-3 in 2021 were excluded as data were collected through a deviant scheme.

Within the environments, data curation was carried out by employing a linear mixed model derived from Eq. 1 due to the heterogeneous designs of field trials:

1 $$Y_{ijk} = \mu_{0} + g_{i} + r_{j} + \left( {rb} \right)_{jk} + \varepsilon_{ijk} ,$$

where µ_0_ is the overall mean of the phenotypic values, *g*_*i*_ is the effect of the *i*^*th*^ genotype, *r*_*j*_ refers to the effect of the *j*^*th*^ replication, *(rb)*_*jk*_ indicates the block effect nested into replication, and *ε*_*ijk*_ is the residual effect. All effects except the overall mean were modeled as random. The resulting variance components were used to estimate the repeatability of each environment as follows:

2 $${\mathrm{repeatability}} = \frac{{\sigma_{g}^{2} }}{{\sigma_{g}^{2} + \frac{{\sigma_{\varepsilon }^{2} }}{n}}},$$

where $\mathit{\upsigma }_{g}^{2}$ refers to the total genotypic variance, $\mathit{\upsigma }_{\varepsilon }^{2}$ refers to the residual variance, and *n* corresponds to the average number of replications per genotype in the respective environment. Raw data of the corresponding trait in an environment with repeatability lower than 0.3 were discarded from the further analysis. It should be noted that, in WP-3, estimates of repeatability are likely to be upwardly biased because only check varieties were replicated, whereas all other entries were unreplicated, resulting in reduced precision of variance component estimation relative to WP-2 and WP-1.

On the curated raw data of each breeding stage, a one-step model was employed to extract the best linear unbiased predictions (BLUPs) and to decompose the variance components for the subsequent estimation of the broad-sense heritability. Due to the differing networks of field tests for each breeding stage, two different models were applied for WP-1, WP-2 (Eq. 3), and WP-3 (Eq. 4):

3 $$Y_{ijkl} = \mu_{0} + g_{i} + e_{j} + \left( {ge} \right)_{ij} + \left( {erb} \right)_{jkl} + \left( {er} \right)_{jl} + \varepsilon_{ijkl}$$

4 $${Y}_{ij}= {\mu }_{0}+ {g}_{i}+ {e}_{j}+ \left({ge}\right)_{ij}+ {\varepsilon }_{ij},$$

where *g*_*i*_ is the effect of the *i*^*th*^ genotype, *e*_*j*_* is* the effect of the *j*^*th*^ environment, (*ge*)_*ij*_ is the genotype-by-environment interaction effect, (*erb*)_*jkl*_ is the block effect nested within replication and environment, (*er*)_*jl*_* is* the replication effect nested within environment, and $\mathit\varepsilon_{ijkl}$ (and $\mathit\varepsilon_{ij}$ ) is the residual effect. For WP-3, the reduced model (Eq. 4) was used to account for the predominantly unreplicated phenotypic data structure without information regarding blocks, while for WP-1 and WP-2, where a partially replicated alpha lattice design was used, a more complex model was employed (Eq. 3). All effects except the overall mean were modeled as random. Outlier correction was performed using method 4 “Bonferroni-Holm with re-scaled median absolute deviation standard residuals” (Bernal-Vasquez et al. 2016).

The broad-sense heritability (*H*^2^) was calculated according to Holland et al. (2002) using the following equation:

5 $$H^{2} = \frac{{\sigma_{g}^{2} }}{{\sigma_{g}^{2} + \frac{{\sigma_{{{\mathrm{ge}}}}^{2} }}{{n_{e} }} + \frac{{\sigma_{\varepsilon }^{2} }}{{n_{{{\mathrm{er}}}} }}}},$$

where $\mathit{\upsigma }_{g}^{2}$ and $\mathit{\upsigma }_{ge}^{2}$ refer to the total genotypic variance of the genotypes and their interaction with the environments, respectively, and $\mathit{\upsigma }_{\varepsilon }^{2}$ refers to the residual variance. The average number of environments is abbreviated as *n*_*e*_, whereas *n*_er_ represents the product of the average number of environments and the average number of replications. For WP-3, heritability estimates may be slightly inflated due to the limited replication of most entries, except for intensively replicated checks across environments.

All the calculations were made using the R Statistical Software (v4.0.1; R Core Team 2021) and the “ASReml” package (v4.1.0; Butler et al. 2019).

### Genotypic data analysis

Genomic profiles were generated for 6,289 out of 13,773 genotypes. Genotyping was performed using 6,719 single nucleotide polymorphisms (SNPs) from the Illumina Infinium 7K SNP array (TraitGenetics, Gatersleben, Germany), which is based on the publicly available 90K SNP array (Wang et al. 2014), and was prioritized on lines with high-quality phenotypic data (WP-1 and WP-2), while early-stage (WP-3) material was genotyped at lower intensity for the purpose of optimization of financial resources. To ensure the quality of the SNP data meets the requirements of the prediction functions, markers and genotypes with missing values exceeding 10% were removed, as were markers with a minor allele frequency below 5% and genotypes that exhibited a significant proportion of heterozygosity. This process resulted in 6,498 high-quality SNP markers for 6,228 genotypes.

Rogers’ distances (Rogers 1972) among all pairs of genotypes were calculated for all subsets and subset combinations. Genotypes with a Rogers’ distance value lower than 0.03 were considered as duplicates. A principal component analysis (PCA) was conducted on the quality-controlled SNP data to ascertain whether the selection processes between breeding stages and years resulted in the formation of population structures, employing the “prcomp” function of the R software (R Core Team 2021). To further assess population structure, an admixture analysis was performed for *K* = 1 to *K* = 30 using the LEA package (Frichot and Francois 2015) in R software (R Core Team 2021), with cross-entropy used to determine the optimal number of ancestral populations. Additionally, a PCA on ancestral populations was conducted to evaluate genetic differentiation among breeding stages. Additionally, the effective population size was estimated following the method of Hill (1981) and the bias correction of Waples (2006).

### Genomic prediction model and scenarios

We used the genomic best linear unbiased prediction (GBLUP) model of the BGLR package (Pérez and de los Campos 2014) in R software (R Core Team 2021) for all prediction scenarios:

6 $$\hat{y}_{i} = \mu + g_{i} + \varepsilon_{i} ,$$

where $\hat{y}_{i}$ is the BLUP of the *i*^*th*^ genotype for the respective trait predicted, *µ* is the overall mean, *g*_*i*_ is the additive genotypic value, and ${\varepsilon }_{i}$ is the residual error for the *i*^*th*^ genotype. The variables of *g* and $\varepsilon$ are expected to be independent. The random vector *g* = (*g*_*1*_*, g*_*2*_*, …, g*_*n*_) follows a normal distribution $g\sim N(0, {\upsigma }_{g}^{2}G)$, where $\mathit{\upsigma }_{g}^{2}$ is the additive genetic variance. The covariance matrix *G* was calculated according to VanRaden (2008) “first method.” For comparison, we also evaluated the Bayesian model “BayesB” from the BGLR package (Pérez and de los Campos 2014) in R software (R Core Team 2021), allowing to account for different SNP effects. Details on the comparison of GBLUP and BayesB are provided in Supplementary Fig. 2.

**Fig. 1:**
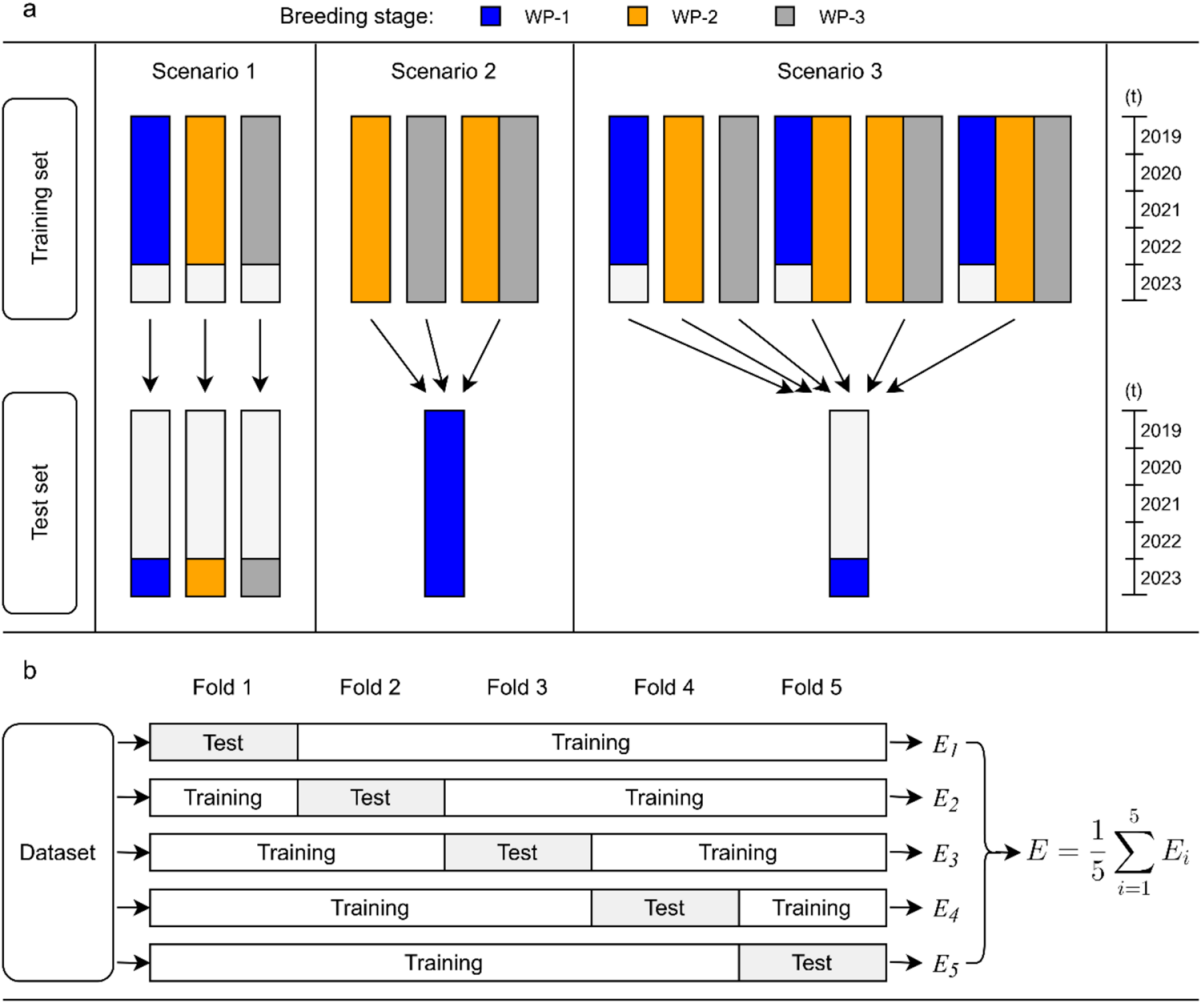
Schematic illustration of three prediction approaches and k-fold cross-validation; as scenarios, (**a**) we applied (i) a prediction of the genotypes of 2023, respectively, for each breeding stage (Scenario 1); (ii) a prediction of the most advanced breeding stage WP-1 with different training set arrangements (Scenario 2); and (iii) a prediction of genotypes from 2023 in breeding stage WP-1 with various compilations of the training set with remaining genotypes of all breeding stages (Scenario 3). For the k-fold cross-validation (**b**) with *k* = 5, each dataset with best linear unbiased predictors is partitioned into k disjoint folds. Then, *k*-1 parts are used for training a model and predicting the remaining part. Iteration of this process for all possible choices of test sets results in prediction abilities per fold (*E*_1_–*E*_*k*_). Prediction ability is reported as a mean per iteration (*E*)

Fivefold cross-validations were performed with 50 runs per breeding stage for each trait (Fig. 1b). The prediction ability was calculated as the correlation between the calculated BLUPs and the predicted values for each run and as a mean across all runs. Subsequently, three genomic prediction scenarios were followed by different constellations of subsets as training sets and test sets (Fig. 1a). In the first scenario, the test set comprises the genotypes tested in 2023, and the training set contains all genotypes tested in the years 2019–2022, separately for each breeding stage. In the second scenario, genotypes of WP-1 across all years served as the test set and the remaining breeding stages and their combination were training sets. For the third scenario, genotypes of WP-1 tested in the year 2023 were assigned to the test set, and a set of data compilations based on genotype data from 2019 to 2022 were used as training sets.

## Results

### Phenotypic data curation and analysis

**Fig. 2:**
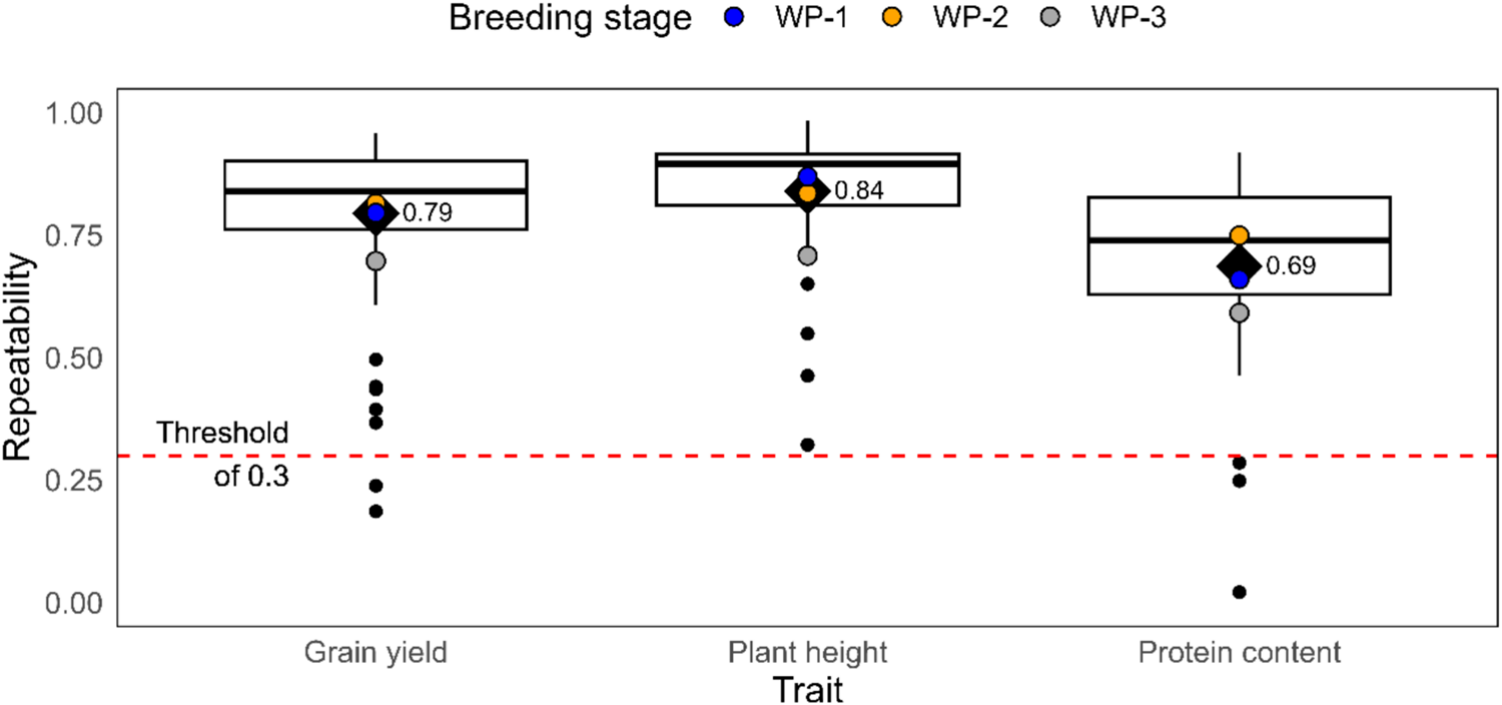
Repeatabilities per environment, i.e., year times location combination, for the traits grain yield, plant height, and protein content. The red-dashed line indicates the threshold for the excluding of raw data

Field trials were conducted in 95 environments, i.e., location times year combinations, to evaluate the winter wheat plant materials for grain yield, plant height, protein content, and yellow rust resistance. The repeatability, i.e., heritability per environment, was used to estimate the data quality. These estimates were moderate to high for all traits (Fig. 2). Repeatabilities ranged from 0.19 to 0.96 for grain yield, from 0.32 to 0.98 for plant height, and from 0.02 to 0.92 for protein content.

To reduce the impact of poor data quality on further analysis, trait data from a small number of environments with repeatability scores below 0.3 were excluded (Supplementary Table 2). While trait-specific thresholds may be expected, this minimal exclusion suggests a negligible impact on overall results. The evaluation of yellow rust resistance was limited to a single replication, precluding the repeatability estimation for this trait.

Variance components estimated using a one-step linear mixed model showed a decrease in genotypic variance ($\mathit{\sigma }_{g}^{2}$) for grain yield, plant height, and protein content from WP-3 to WP-1, reflecting the selection process in ongoing breeding (Table 1). In contrast, the interaction variance of genotypes and environments ($\mathit{\sigma }_{ge}^{2}$) remained relatively constant across all three breeding stages for all traits. Broad-sense heritability estimates were high for WP-1 (0.82, 0.92, 0.74, and 0.83 for grain yield, plant height, protein content, and yellow rust resistance, respectively), moderate for WP-2 (0.72, 0.84, 0.65, and 0.80), and lower for WP-3 (0.33, 0.51, 0.43, and 0.16). The lower heritabilities in WP-3 result from the limited number of environments and the fact that $\mathit{\upsigma }_{ge}^{2}$ was approximated only through repeated check varieties, as most WP-3 entries were selection candidates without replication. Despite this, our analysis accounted for this limitation and confirmed the overall data quality.

**Table 1:** Variance components from the one-step analysis for the traits grain yield, plant height, protein content, and yellow rust resistance for each breeding stage

Trait	Breeding stage	$\mathit{\sigma }_{g}^{2}$	$\mathit{\sigma }_{ge}^{2}$	$\mathit{\sigma }_{\varepsilon }^{2}$	*H*^2^
Grain yield	WP-1	6.22	13.14	6.51	0.82
	WP-2	12.69	14.59	12.78	0.72
	WP-3	18.29	15.16	21.65	0.33
Plant height	WP-1	18.48	7.15	4.44	0.92
	WP-2	23.71	5.82	11.13	0.84
	WP-3	24.57	11.07	13.68	0.51
Protein content	WP-1	0.14	0.09	0.08	0.74
	WP-2	0.16	0.14	0.16	0.65
	WP-3	0.17	0.15	0.19	0.43
Yellow rust	WP-1	0.86	0.00	1.23	0.83
	WP-2	1.06	0.40	0.77	0.80
	WP-3	0.32	0.38	1.44	0.16

$\mathit{\sigma }_{g}^{2}$ = variance of genotypes, $\mathit{\sigma }_{ge}^{2}$ = variance of genotype and environment interaction, $\mathit{\sigma }_{\varepsilon }^{2}$ = variance of residuals, and *H*^2^ = broad-sense heritability

### Absence of population structure according to breeding stages

**Fig. 3:**
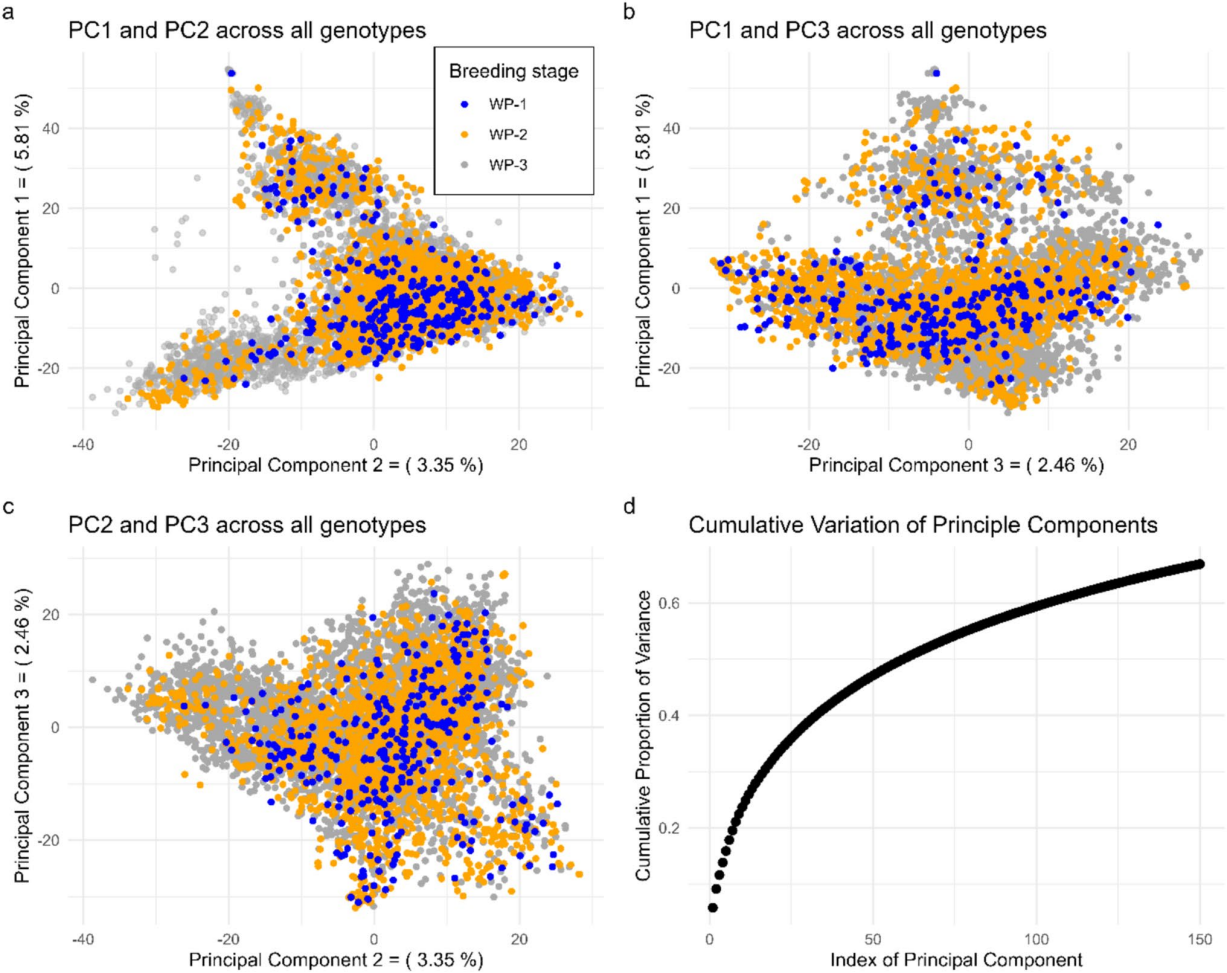
Plot (**a**-**c**) of first, second, and third principal components (PC1, PC2, PC3) for all genotypes based on 6,498 SNP markers and (**d**) cumulative variance explained by the first 150 principal components

Principal component analysis (PCA) was performed to identify the underlying structure within the breeding population. The plot of the first three principal components showed that genotypes from all three breeding stages were widely scattered, with no clear clustering (Fig. 3a–c). Cumulatively, the first 150 principal components explained around 70% of the variation (Fig. 3d). The first, second, and third principal components accounted for only 5.81%, 3.35%, and 2.46% of the molecular variation, respectively.

Admixture analysis revealed no distinct genetic clustering among breeding stages across all tested *K* values, with no pronounced elbow in the cross-entropy curve (Supplementary Fig. 3). Similarly, PCA on ancestry proportions showed a continuous distribution of genotypes across breeding stages (Supplementary Fig. 3), indicating minimal genetic differentiation and suggesting no substantial effect on the prediction ability. In conclusion, no genetically distinct subpopulations were observed.

The influence of selection on genetic variation was assessed by estimating the effective population size within but also across different breeding stages and years. While subset sizes ranged from 310 to 6,209 genotypes, the effective population size varied between 37.08 and 45.69 (Table 2). The ratio of subset sizes to effective population size varied from 8.36 to 135.89, with the subset size being the primary driver of this ratio, as effective population size remained relatively constant. Mean pairwise Rogers’ distances for genotypes within each subset and subset combination averaged 0.343 (± 0.04) (Table 3).

**Table 2:** Effective population size, number of genotypes, and ratio between the size of the subset (combination) and the effective population size for each subset or subset combination

Parameter	WP-1	WP-2	WP-3	WP-1 + WP-2	WP-2 + WP-3	WP-1 + WP-2 + WP-3
*N*_*e*_	37.08	44.53	44.67	45.05	45.35	45.69
*n*	310	1924	5642	2006	6127	6209
*n*/*N*_*e*_	8.36	43.21	126.30	44.53	135.10	135.89

**Table 3:**
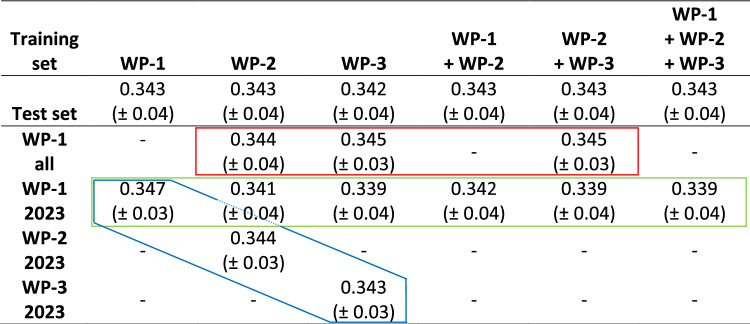
Mean pairwise Rogers’ distances with respective standard deviations for each subset and between each test set and training set for all prediction approaches: (i) within each breeding stage with year-specific genotype assignments (blue box); (ii) across breeding stages (red box); and (iii) for genotypes from 2023 WP-1 (green box)

The mean Rogers’ distances between the subsets ranged from 0.339 to 0.347, closely matching the values observed within each subset. Further analysis revealed a very low correlation between mean pairwise Rogers’ distance and the prediction ability (Supplementary Fig. 4).

### Genomic predictions

**Fig. 4:**
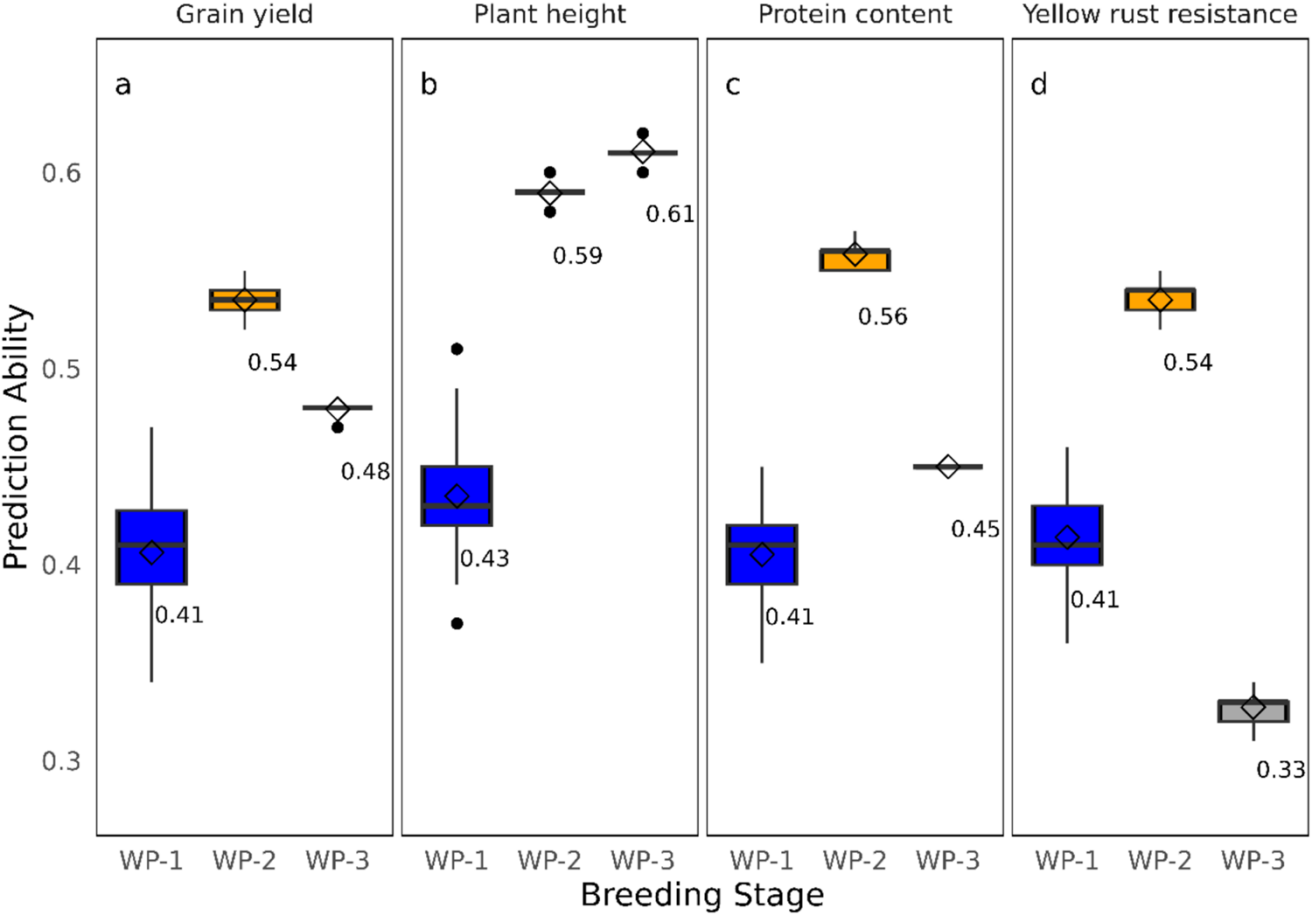
Mean prediction abilities from fivefold cross-validation for the analyzed traits: (**a**) grain yield, (**b**) plant height, (**c**) protein content, and (**d**) yellow rust resistance, reported separately for each breeding stage

Fivefold cross-validation was used to assess the prediction ability within each breeding stage (Fig. 1b). The mean prediction abilities for grain yield were 0.41 for WP-1, 0.54 for WP-2, and 0.48 for WP-3 (Fig. 4). For plant height, the prediction abilities were 0.43, 0.59, and 0.61, for protein content they were 0.41, 0.56, and 0.45, and for yellow rust resistance they were 0.41, 0.54, and 0.33, for WP-1, WP-2, and WP-3, respectively. A comparison of the GBLUP model with the BayesB method showed that, for most traits and breeding stages, BayesB can have similar prediction ability as the GBLUP. Interestingly, for the WP-2 stage, a consistent increase in the average prediction ability was observed for all four traits (Supplementary Fig. 2).

**Fig. 5:**
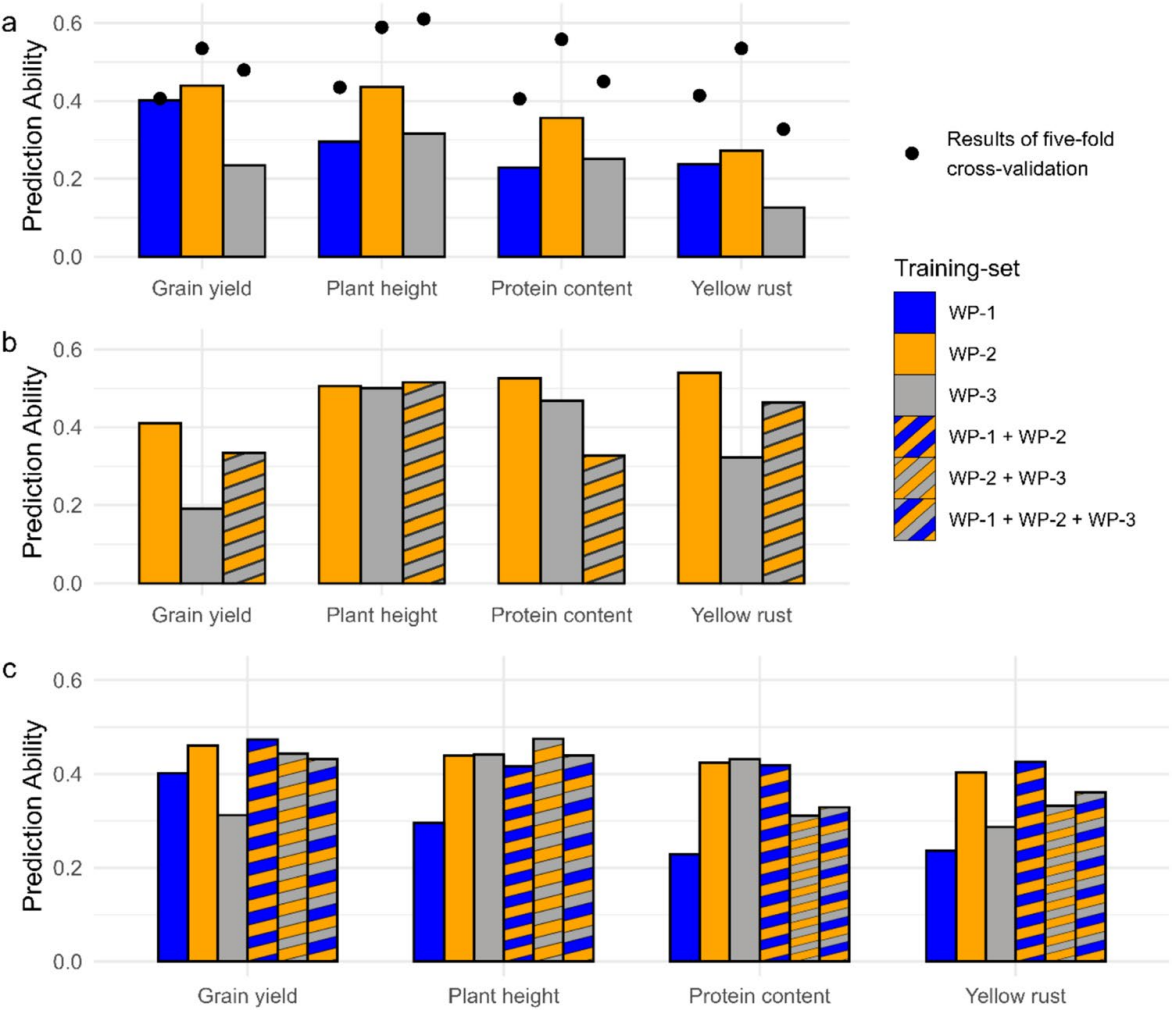
Prediction abilities for three approaches: (**a**) The test sets consist of genotypes from 2023 of a specific breeding stage, while the training sets are genotypes from the same breeding stage using 2019–2022 data. Bars indicate the prediction ability when genotypes of 2023 are used as a test set. The black points represent the results of fivefold cross-validation within each breeding stage. (**b**) The test set includes data from the entire WP-1 breeding stage, while the training sets comprise combinations of genotypes from WP-2 and/or WP-3. (**c**) The test set includes genotypes of WP-1 from the year 2023, while the training sets consist of the remaining genotypes, both within and across breeding stages

Prediction abilities were further evaluated using three alternative approaches (Fig. 1a): The first scenario predicted the performance of genotypes tested in 2023 using the data within breeding stages from 2019 to 2022 as a training set. For this scenario, grain yield prediction abilities were 0.40, 0.44, and 0.23 for WP-1, WP-2, and WP-3, respectively (Fig. 5a). For plant height, they were 0.30, 0.44, and 0.32; for protein content, 0.23 (WP-1), 0.36 (WP-2), and 0.25 (WP-3); and for yellow rust, 0.24 (WP-1), 0.27 (WP-2), and 0.13 (WP-3).

In the second scenario, all WP-1 genotypes were predicted using WP-2, WP-3, and a combined WP-2 + WP-3 training set (Fig. 5b). The test set consisted of 310 genotypes, with training sets containing 1696, 5475, and 5899 genotypes for WP-2, WP-3, and WP-2 + WP-3, respectively. For grain yield, the highest prediction ability (0.41) was achieved with WP-2 as the training set, followed by 0.33 for WP-2 + WP-3, and 0.19 for WP-3. For plant height, the combined training set (WP-2 + WP-3) yielded the highest prediction ability (0.52), followed by WP-2 (0.51) and WP-3 (0.50). In contrast, for protein content, WP-2 gave the highest prediction ability (0.53), with WP-3 at 0.47 and WP-2 + WP-3 at 0.33. Yellow rust resistance followed a similar trend to grain yield, with WP-2 (0.54), WP-3 (0.32), and WP-2 + WP-3 (0.46). In summary, the second approach demonstrated that optimizing the composition of the training population to maximize prediction abilities varied across traits.

In the third approach, prediction abilities were assessed for WP-1 genotypes tested in 2023, using various training sets: WP-1 from previous years, WP-2, WP-3, and combinations of these stages (WP-1 + WP-2, WP-2 + WP-3, and WP-1 + WP-2 + WP-3) (Fig. 5c). The test set contained 79 genotypes. Training set sizes ranged from 231 genotypes for WP-1 (excluding 2023) to 6,130 for the combined WP-1 + WP-2 + WP-3 dataset. For grain yield, the highest prediction ability (0.47) was achieved with WP-1 + WP-2 as the training set, followed by 0.46 for WP-2, 0.44 for WP-2 + WP-3, 0.43 for WP-1 + WP-2 + WP-3, 0.40 for WP-1, and 0.31 for WP-3. For plant height, WP-2 + WP-3 resulted in the highest prediction ability (0.48), followed by 0.44 for WP-1 + WP-2 + WP-3, WP-2, and WP-3, and 0.42 for WP-1 + WP-2, and WP-1 at 0.29. Regarding the trait protein content, the prediction abilities were 0.43 (WP-3), 0.42 (WP-1 + WP-2, and WP-2), 0.33 (WP-1 + WP-2 + WP-3), 0.32 (WP-2 + WP-3), and 0.23 (WP-1). Prediction abilities for yellow rust resistance were more variable, with values of 0.43 (WP-1 + WP-2), 0.40 (WP-2), 0.36 (WP-1 + WP-2 + WP-3), 0.33 (WP-2 + WP-3), 0.29 (WP-3), and 0.24 (WP-1).

## Discussion

This study aimed to leverage genotypic and phenotypic data from a winter wheat breeding program across various breeding stages to explore the optimal composition of training sets for genomic predictions for key traits. Data curation, including filtering environments with low repeatabilities, resulted in heritability estimates ranging from high for WP-1 to moderate for WP-2 and low for WP-3 (Table 1). These estimates reflect the allocation of resources across breeding stages and align with findings from previous wheat studies (Isidro et al. 2015; Norman et al. 2017; Schopp et al. 2017; Ficht et al. 2023), providing a solid data foundation for investigating the potential of genomic selection. However, the lower precision of repeatability and heritability estimates in WP-3, resulting from variance components estimation based on replicated check varieties, should be considered when interpreting the results. In addition, the genotyping strategy in this study deliberately prioritized genotypes with high-quality and comprehensive phenotypic data (WP-1 and WP-2), resulting in lower genotyping intensity in early breeding stages (WP-3). This targeted approach reflects common practice in commercial breeding, where resources are strategically allocated to maximize the utility of the training data. While this may introduce a degree of selection bias, it also enhances the overall reliability of genomic predictions by focusing on phenotypic data of higher precision.

The accuracy of genomic prediction was evaluated for subsets composed of data from three breeding stages and combinations of these stages. The prediction ability, assessed with fivefold cross-validation within the breeding stage per trait, showed notable differences (Fig. 4) directly linked to differences in heritabilities and the $\frac{n}{{N}_{e}}$ ratio (Table 2). The observed variation in the prediction ability across subsets highlights not only the role of heritability but underscores the complementary influence of training set size and the $\frac{n}{{N}_{e}}$ ratio, as larger training populations can improve the accuracy of genomic predictions. These findings align with previous studies (Meuwissen 2009; Combs and Bernardo 2013; Norman et al. 2018; Zhao et al. 2021), which found a positive association of heritability, training set size, and the $\frac{n}{{N}_{e}}$ ratio with the prediction ability.

A comparison of the GBLUP model with BayesB showed no consistent improvement in the prediction ability for breeding stages WP-1 and WP-3 (Supplementary Fig. 2), reinforcing the suitability of GBLUP for practical breeding applications. In the breeding stage WP-2, the BayesB model exhibited higher prediction accuracy across all four traits, which is somewhat unexpected. This likely reflects the change in family structure and increased selection pressure in WP-2, where certain quantitative trait loci (QTLs) may exert larger effects. Under such conditions, BayesB, which can capture non-infinitesimal effects, may outperform GBLUP, which assumes equal marker variances. Future analyses, particularly the genome-wide association study (GWAS) for WP-2, may provide deeper insights into the underlying reasons for this observation.

Another factor potentially driving the prediction ability is the relatedness between training and test populations (Asoro et al. 2011; Clark et al. 2012; Isidro et al. 2015; Edwards et al. 2019). The mean pairwise Rogers’ distance was used to assess the relatedness of genotypes within and between all subsets, resulting in a mean value of 0.343 (± 0.04) for all subsets, with comparable values ranging from 0.339 to 0.347 between subsets (Table 3). This consistency suggests no pronounced differences in the average relatedness of genotypes either within each subset or between subsets for each prediction scenario. To further quantify the impact of genetic relatedness and the prediction ability, we evaluated the correlation between Rogers’ distance and prediction performance. The low R^2^ value of 0.054 (p = 0.114) indicates that relatedness was not a primary determinant of the prediction ability in this study (Supplementary Fig. 2). In summary, the differences in prediction ability observed across the subsets in our study are most likely driven by differences in heritabilities, the number of individuals, and the $\frac{n}{{N}_{e}}$ ratio among the different training sets.

The potential pitfall of overestimating the prediction ability when applying fivefold cross-validation becomes apparent when using genotypes evaluated in 2023 of a specific breeding stage as a test set but excluding them from the training sets (scenario 1, Fig. 5a). For WP-3, we observed significant differences in prediction abilities between scenario 1 and fivefold cross-validation. The latter is likely to be overestimated due to confounded genotype effects, with interaction effects between genotypes and environments, as the trials were conducted in only one environment with sole replications for check varieties. This highlights how genotype-by-environment interactions can lower the prediction ability even with increased population size, particularly when limited environmental replications occur. Our findings emphasize the need for test populations from independent environments to obtain robust estimates of genomic prediction abilities.

The high heritability of the WP-1 population makes it a particularly suitable test set for detecting subtle differences between different training populations (scenario 2, Fig. 5b). Contrary to expectations, WP-2 as the training set outperformed the combined training set of WP-2 + WP-3 for complex traits such as grain yield, protein content, and yellow rust resistance (Fig. 5b), despite its smaller size. This can be attributed to the considerably lower heritability of WP-3 compared to WP-2, which cannot be compensated by the increase in population size. Additionally, the genetic effects in WP-3 may be confounded with interaction effects between genotypes and environments, potentially limiting its usefulness in predicting WP-1 genotypes. In contrast, for plant height—a trait with high heritability even in WP-3—a combined training set of WP-2 + WP-3 resulted in the highest prediction ability. From these results, we conclude that WP-2 serves as a strong basis for the training set, and adding WP-3 data can enhance the prediction ability if its heritability is reasonably high, e.g., greater than 0.5.

To assess whether severely adverse effects arise when data from all breeding stages are integrated into a training dataset, we used only the 2023 data from WP-1 as a test population (scenario 3, Fig. 5c). For plant height and, surprisingly, grain yield, filtering the data did not lead to substantial changes in prediction accuracy compared to using data from WP-1, WP-2, and WP-3. This contrasts with previous studies (e.g., He et al. 2016), which reported increased prediction accuracy when including genotypes tested in multiple environments. However, for protein content and yellow rust susceptibility, integrating WP-1 and WP-2 data while excluding WP-3 led to significantly higher prediction accuracy than using all data. This improvement is likely due to significant genotype-by-year interactions for these traits.

In conclusion, for complex traits such as grain yield, protein content, and yellow rust, where genotype-by-environment interaction plays a role, omitting WP-3 from the training set combinations is a good option, and the optimum prediction ability can be obtained by combining WP-1 and WP-2. However, for less complex traits such as plant height, a more extensive training set positively impacted the prediction ability, particularly when the heritability levels were comparable between the breeding stages.

Our findings demonstrate that breeders must carefully balance the trade-off between population size and heritability when selecting training sets. Furthermore, integrating genomic selection into breeding pipelines requires a strategic approach to increase efficiency. By predicting ahead, breeders can make earlier selection decisions and potentially reduce the need for extensive field trials while maintaining genetic gain. Also, genomic selection can complement traditional selection methods by incorporating valuable selection information, even when data quality is limited.

## Conclusion

In this study, we explored the implementation of genomic prediction using data from a breeding program. Predicting ahead enables earlier selection, reduces field testing resources, and facilitates faster breeding decisions. Integrating genomic selection into breeding pipelines allows breeders to strategically allocate resources and optimize the use of phenotypic data of different qualities. The high relevance of WP-2, in particular, highlights the need for a balanced compromise between heritability and population size for this sample size. To significantly increase the population size without sacrificing heritability, an integrated analysis across different breeding programs would be a promising alternative. The carefully curated genotypic and phenotypic data represent an important first step in this direction. Interoperability is facilitated by embedding these data into a broader data ecosystem (Lell et al. 2025), enabling genomic predictions to be considered beyond the data silos of individual breeding programs.

## Supplementary Information

Below is the link to the electronic supplementary material.

Supplementary file1 (DOCX 496 KB)

## Data Availability

The datasets analyzed during the current study are available from the corresponding author upon reasonable request.
