# Supplementary material for: Integrating genomic predictions into an applied Central European wheat breeding program
Source: Theor Appl Genet. 2026 Feb 13;139(2):69. doi: 10.1007/s00122-026-05175-z (PMC12904978; doi:10.1007/s00122-026-05175-z)
Supplement: Supplementary file 1 — Supplementary file1 (DOCX 496 KB) [file 122_2026_5175_MOESM1_ESM.docx]

Integrating genomic predictions into an applied
Central European wheat breeding program

Journal: Theoretical and Applied Genetics

Lars Erik Thomsen^a#^; ORCiD: 0009-0003-7405-5338,
E-mail: lars.e.thomsen@agrar.uni-giessen.de; Tel.: +49 0641 9937547.

Yusheng Zhao^a^; ORCiD: 0000-0001-6783-5182;
E-mail: zhao@ipk-gatersleben.de; Tel.: +49 39482 5-404.

Ulrike Avenhaus^b^; ORCiD: 0000-0001-9711-1409;
E-mail: U.Avenhaus@wvb-eckendorf.de; Tel.: +49 5208 9125-24.

Jochen Christoph Reif^a*^; ORCiD: 0000-0002-6742-265X;
E-mail: reif@ipk-gatersleben.de; Tel.: +49 39482 5-840.

Ravindra Reddy Gundala^a^; ORCiD: 0000-0002-3877-3645;
E-mail: gundala@ipk-gatersleben.de; Tel.: +49 39482 5-843.

^a^ Department of Breeding Research, Leibniz Institute of Plant Genetics and Crop Plant Research (IPK), Corrensstraße 3, 06466 Seeland OT Gatersleben, Germany.

^b^ W. von Borries-Eckendorf GmbH & Co. KG, Hovedisser Straße 94, 33818 Leopoldshöhe, Germany.

* Corresponding author. E-mail: reif@ipk-gatersleben.de (J.C. Reif).

Present Address:

^#^ Department of Biometry and Population Genetics, Justus Liebig University Gießen, Heinrich-Buff-Ring 26, 35392 Gießen, Germany.

# Supplementary Information

## Supplementary Figures


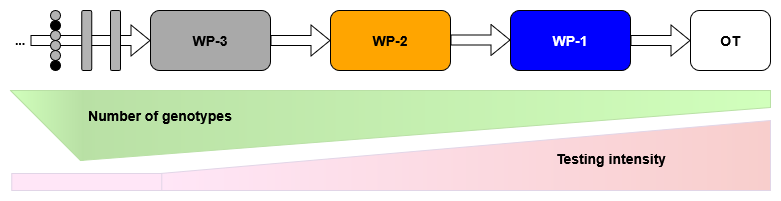


**Supplementary Figure 1** Simplified scheme of the breeding pipeline illustrating the decline in the number of genotypes due to the process of selection and the increase in testing intensity through conducted environments and replications in field trials. Preceding steps of the breeding scheme for genotype development are conducted before breeding stages WP-3 to WP-1 follow with field tests for genotype evaluation and potential variety testing at the Federal Plant Variety Office. Prior to the yield trials, breeding lines (represented as bars) are established from individual plants (depicted as dots) originating from field nursery trials and propagations in green houses. Genotyping intensity follows phenotyping quality. OT = Official Tests

**
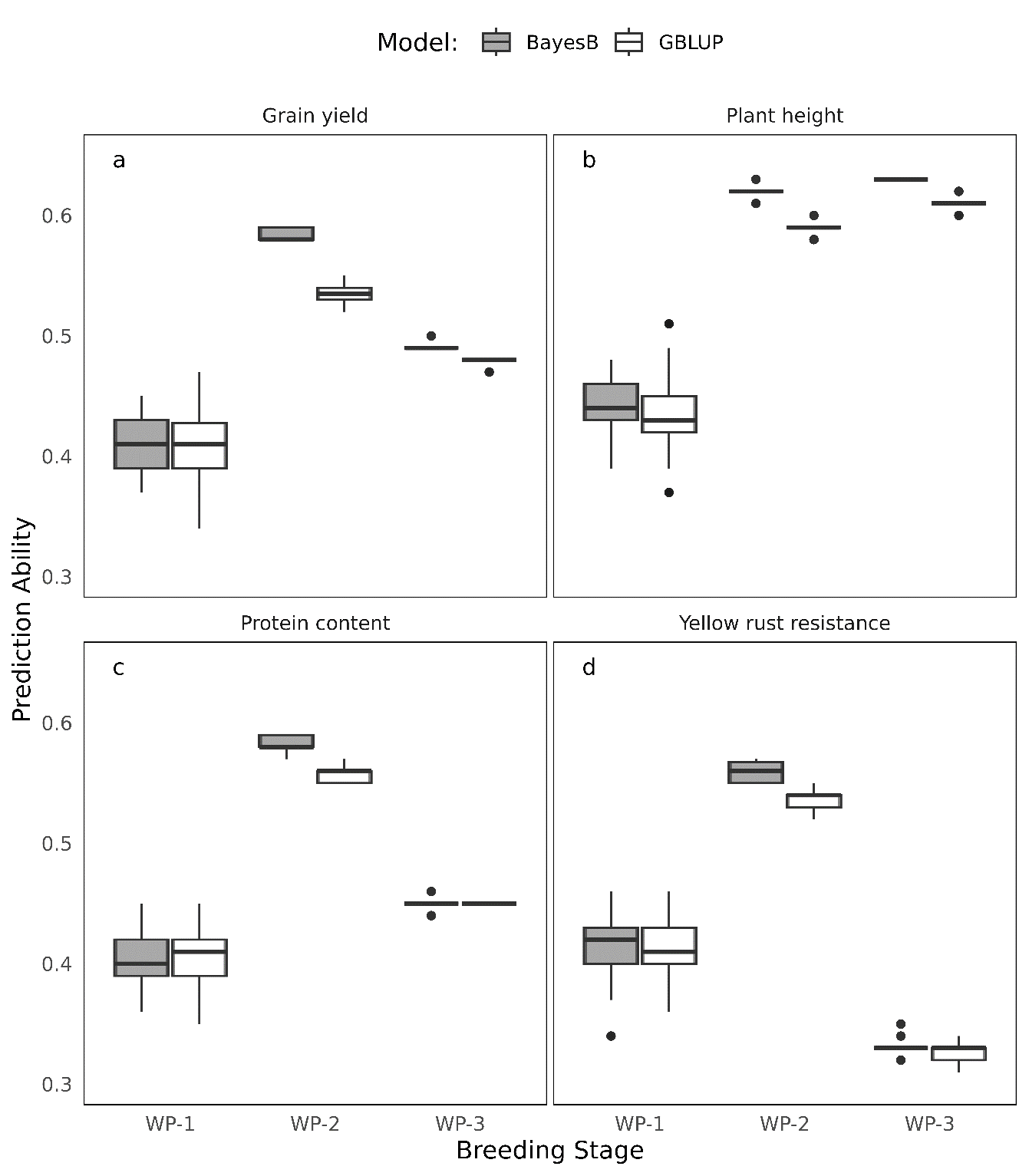
**

**Supplementary Figure 2** Mean prediction abilities from five-fold cross-validation comparing genomic best linear unbiased predictor (GBLUP) model with BayesB model for the analyzed traits: (a) grain yield, (b) plant height, (c) protein content, and (d) yellow rust resistance, reported separately for each breeding stage


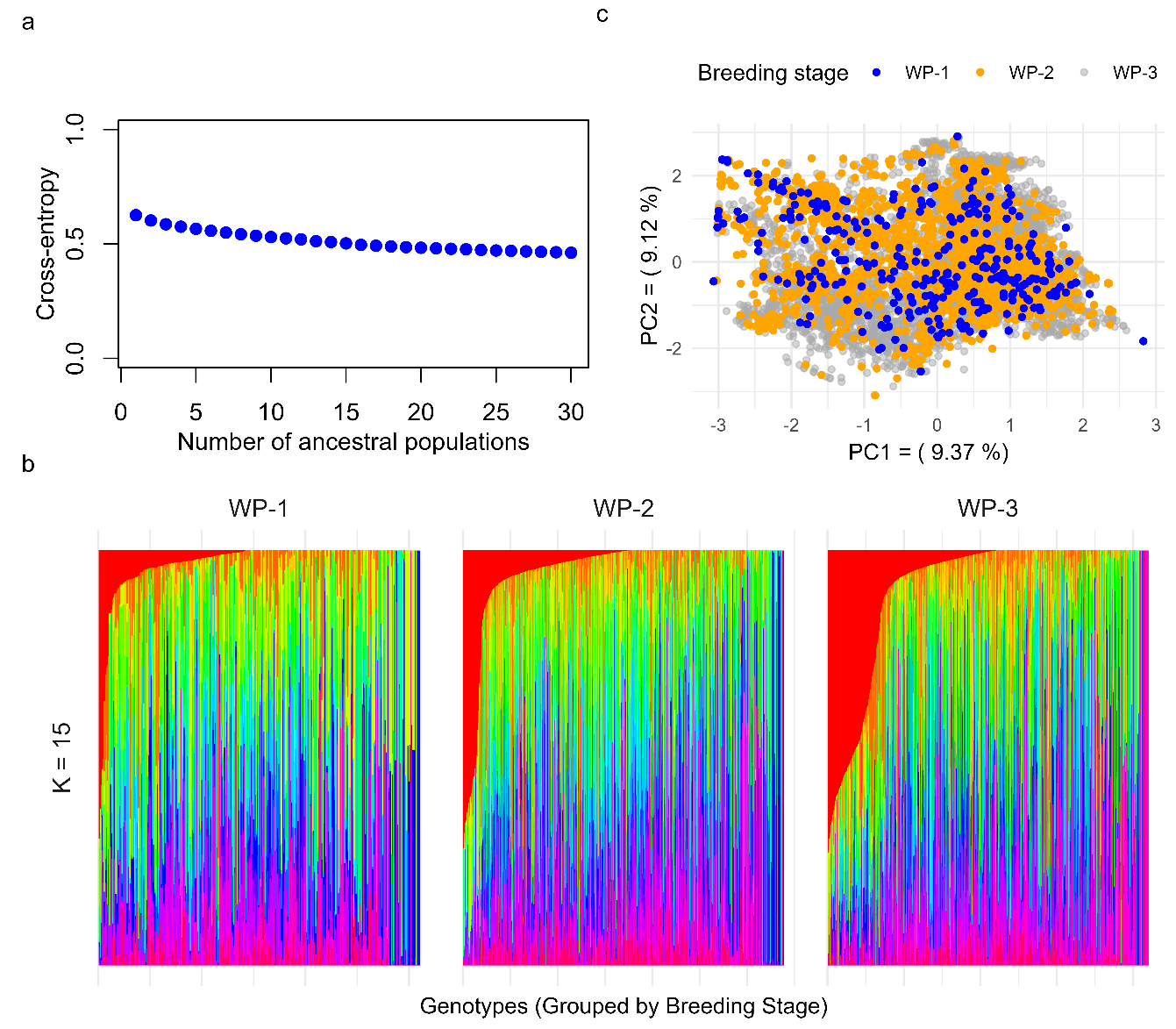


**Supplementary Figure 3** Population structure inferred using admixture analysis: (a) Cross-entropy values for K = 1 to K = 30 from admixture analysis. (b) Estimated population structure with K = 15 ancestral populations using R-package LEA in 6,228 wheat lines from three breeding stages genotyped with 6,498 SNP markers. Each individual is represented by a vertical (100 %) stacked column indicating the proportions of ancestry in K = 15 constructed ancestral populations. (c) First and second principle component (PC1, PC2) based on ancestral proportions of K = 15 ancestral populations


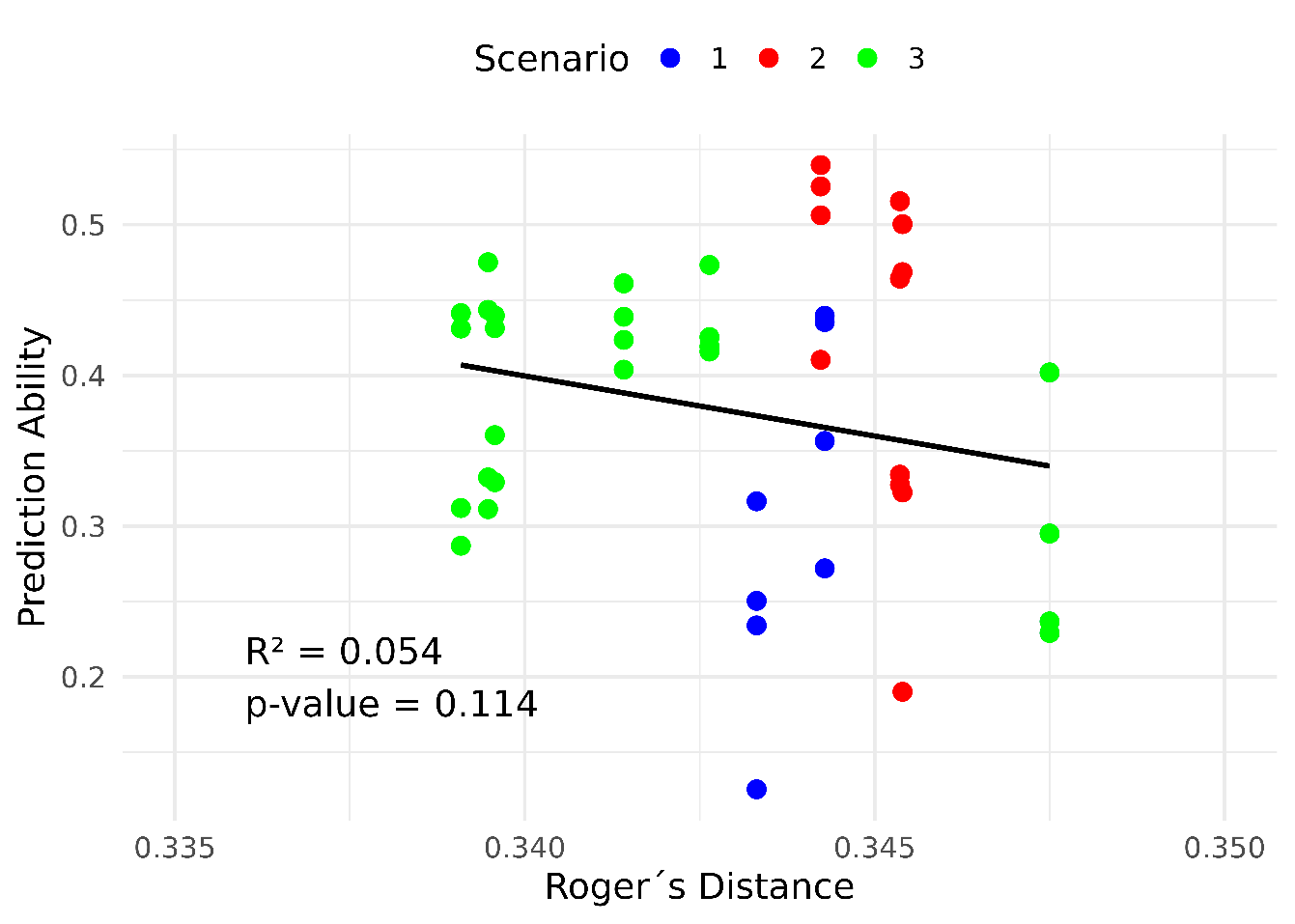


**Supplementary Figure 4** Relationship between Roger´s Distance and Prediction Ability across prediction scenarios

## Supplementary Tables

**Supplementary Table 1** Trial design and Experimental Treatments in Phenotypic data set

| **Breeding stage** | **Treatment** | **No. of env. across years** | **Experimental design** | **Traits assessed** |
| --- | --- | --- | --- | --- |
| **WP-1** | Treated | 61 | Partial replicated  design | Grain yield, Plant height, Protein content |
|  | Untreated | 29 | Unreplicated design | Yellow rust resistance |
| **WP-2** | Treated | 21 | Partial replicated  design | Grain yield, Plant height, Protein content |
|  | Untreated | 25 | Unreplicated design | Yellow rust resistance |
| **WP-3** | Treated | 5 | Unreplicated design (only checks repl.) | Grain yield, Plant height, Protein content |
|  | Untreated | 5 | Unreplicated design | Yellow rust resistance |

**Supplementary Table 2** Raw data of environments excluded from further analysis resulting from quality control

| **Location** | **Breeding stage** | **Year** | **Trait** | **Repeatability** |
| --- | --- | --- | --- | --- |
| **02** | WP-1 | 2019 | yield | 0.24 |
| **13** | WP-1 | 2022 | yield | 0.19 |
| **13** | WP-1 | 2022 | protein content | 0.02 |
| **10** | WP-1 | 2023 | protein content | 0.25 |
| **14** | WP-1 | 2023 | protein content | 0.29 |
